# “Kelene” (Pure Chloride of Ethyl)

**Published:** 1909-10

**Authors:** 


"KELENE" (pure chloride of ethyl). It will interest the profession to learn that in addition to their well known Automatic Glass Spraying Tubes, the manufacturers (Fries Bros., 92 Reade Street, N. Y.), are also furnishing sealed tubes containing 3 c.c. and 5 c.c.

These sealed tubes are designed to fit any inhaler the physician may have purchased for the administration of mixtures.

Every practitioner knows that the good qualities of these mixtures are in proportion to the amount of pure chloride of ethyl they contain and the small percentage of admixtures is a detriment.

The mixing of two or more products with different boiling points can not help but act differently according to the various conditions liable to prevail.

"Kelene" gives quick and effectual results and on account of its purity (absolutely assured by *new* glass tubes) is considered far safer than any other brands of chloride of ethyl, or any of the so-called similar anesthetics. A trial will determine its many advantages.

